# Ethidium tetra­phenyl­borate aceto­nitrile disolvate

**DOI:** 10.1107/S2414314622009518

**Published:** 2022-10-11

**Authors:** Runa Shimazaki, Masaaki Sadakiyo

**Affiliations:** a Department of Applied Chemistry, Faculty of Science Division I, Tokyo University of Science, 1-3 Kagurazaka, Shinjuku-ku, Tokyo, 162-8601, Japan; University of Aberdeen, Scotland

**Keywords:** crystal structure, ethidium salt, tetra­phenyl­borate

## Abstract

In the title solvated salt, (C_21_H_20_N_3_){B(C_6_H_5_)_4_}·2CH_3_CN (systematic name 3,8-di­amino-5-ethyl-6-phenyl­phenanthridin-5-ium tetra­phenyl­borate aceto­nitrile disolvate), the dihedral angle between the tricyclic fused ring system (r.m.s. deviation = 0.021 Å) and the pendant phenyl group of the ethidium cation is 84.91 (7)°. The {B(C_6_H_5_)_4_}^−^ anion has a typical tetra­hedral structure. The aceto­nitrile solvent mol­ecules do not accept hydrogen bonds from the –NH_2_ groups of the ethidium ions.

## Abstract 2

In the title structure, the ethidium cations show a planar structure without dimerization.

## Graphical abstract

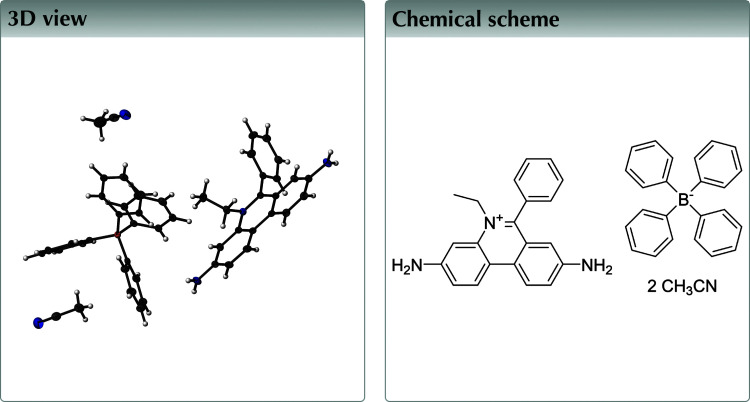

## Structure description

The bromide salt of the C_21_H_20_N_3_^+^ ethidium cation is an important chemical in various research fields such as biochemistry (Chen *et al.*, 2000 ▸) and materials chemistry (Ma *et al.*, 2016 ▸). In this work, the crystal structure of a new solvated ethidium salt, (C_21_H_20_N_3_){B(C_6_H_5_)_4_}·2CH_3_CN, including a bulky anion, tetra­phenyl­borate, was determined.

**Figure 1:**
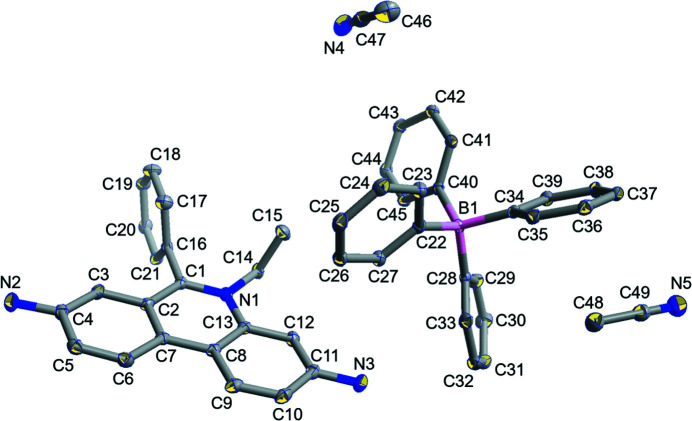
The mol­ecular structure of (C_21_H_20_N_3_){B(C_6_H_5_)_4_}·2CH_3_CN with displacement ellipsoids drawn at the 50% probability level. Hydrogen atoms are omitted for clarity.

One ethidium cation, one tetra­phenyl­borate anion and two aceto­nitrile mol­ecules exist in a unit cell as the crystallographically independent components (Fig. 1 ▸). The ethidium cation shows an almost planer structure in the π-conjugated part (r.m.s. deviation for C1–C13/N1 = 0.021 Å), while that part was observed as a puckered structure in ethidium hepta­fluoro­butrate (Shimazaki *et al.*, 2022 ▸). The dihedral angle between the tricyclic fused ring system and the pendant C16–C21 phenyl group is 84.91 (7)°. The tetra­phenyl­borate anion has a typical tetra­hedral structure around the B atom and aceto­nitrile solvent mol­ecules are incorporated in the voids of the structure.

**Figure 2:**
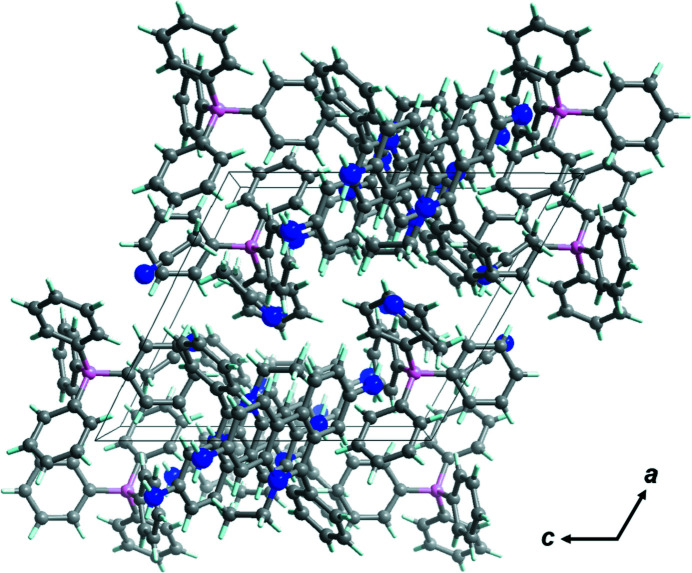
Packing of the title compound viewed along the *b-*axis direction.

**Figure 3:**
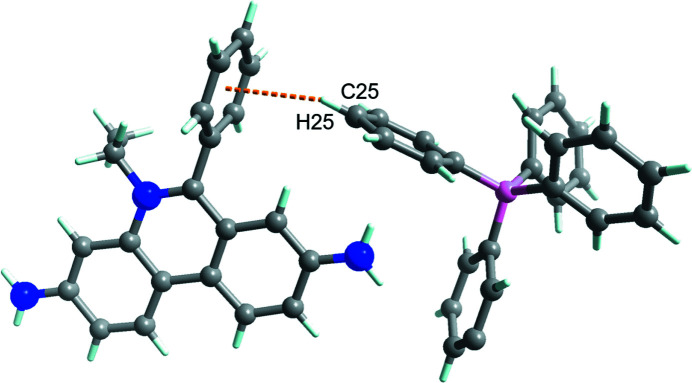
A short contact between the components with a C—H⋯π inter­action, exemplified by ethidium and tetra­phenyl­borate.

In the extended structure of the title compound, the ethidium cations do not show a π–π-dimerized structure [*i.e.*, the closest *Cg*⋯*Cg* separation between the ethidium cations is 4.4611 (5) Å], which is a clear difference between the title structure and that of ethidium hepta­fluoro­butrate (Shimazaki *et al.*, 2022 ▸). There are no hydrogen bonds between the hydrogen-bonding donor (*i.e.*, –NH_2_ on the ethidium cation) and acceptor (*i.e.*, –CN on the aceto­nitrile unit) sites in the title salt but two N—H⋯π inter­actions occur (Fig. 2 ▸, Table 1 ▸). There are also a number of short C—H⋯π inter­actions (Tsuzuki *et al.*, 2000 ▸) involving aromatic rings of both anion and cation as acceptors (Fig. 3 ▸), which are presumably one of the main cohesive inter­actions in this crystal structure.

## Synthesis and crystallization

Firstly, silver tetra­phenyl­borate was synthesized according to a previous report (Borodin *et al.*, 2021 ▸). An aqueous solution (2.7 ml) of sodium tetra­phenyl­borate (182 mg, 0.53 mmol) was mixed with an aqueous solution (1.1 ml) of silver(I) nitrate (181 mg, 1.06 mmol). The mixture was then stirred for 1 h at room temperature. The resulting precipitate, silver(I) tetra­phenyl­borate, was collected by filtration. Next, the obtained powder of silver(I) tetra­phenyl­borate was dissolved in DMSO (50 ml), and then a DMSO solution (10 ml) of ethidium bromide (184 mg, 0.47 mmol) was added to this solution. After stirring for 18 h at room temperature, the resulting precipitate was removed by centrifugation. After adding water to the remaining solution, the resulting powder was collected by centrifugation. The sample powder was again dissolved in acetone, and the insoluble precipitate was removed. The crude powder of the target compound was obtained by vacuum concentration of the remaining solution (185 mg, 0.29 mmol, yield 62%). The title crystal was prepared by recrystallization through slow evaporation (3 days at room temperature) of a solution of the crude powder (30 mg) dissolved in a mixed solvent (4 ml, CH_3_CN: H_2_O = 9: 1).

## Refinement

Details of crystal data, data collections, and structure refinements are shown in Table 2 ▸.

## Supplementary Material

Crystal structure: contains datablock(s) I, global. DOI: 10.1107/S2414314622009518/hb4412sup1.cif

Structure factors: contains datablock(s) I. DOI: 10.1107/S2414314622009518/hb4412Isup2.hkl

Supporting information file. DOI: 10.1107/S2414314622009518/hb4412Isup3.cdx

Supporting information file. DOI: 10.1107/S2414314622009518/hb4412Isup4.cml

CCDC reference: 2209658

Additional supporting information:  crystallographic information; 3D view; checkCIF report

## Figures and Tables

**Table 1:** Hydrogen-bond geometry (Å, °) *Cg*1–*Cg*5 and *Cg*8 are the centroids of the C22–C27, C28–C33, C34–C39, C40–C45, N1/C1/C2/C7/C8/C13 and C16–C21 rings, respectively.

*D*—H⋯*A*	*D*—H	H⋯*A*	*D*⋯*A*	*D*—H⋯*A*
N2—H2⋯*Cg*1^i^	0.88	2.45	3.303 (2)	165
N2—H2*A*⋯*Cg*4^i^	0.88	2.62	3.3297 (18)	139
C21—H21⋯*Cg*3^ii^	0.95	2.75	3.685 (2)	167
C25—H25⋯*Cg*8^i^	0.95	2.79	3.632 (2)	148
C36—H36⋯*Cg*5^iii^	0.95	2.81	3.3593 (18)	118
C48—H48⋯*Cg*2	0.98	2.78	3.609 (2)	143
C48—H48*A*⋯*Cg*3	0.99	2.61	3.488 (2)	149

Symmetry codes: (i) 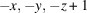 ; (ii) 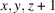 ; (iii) 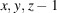 .

**Table 2:** Experimental details

Crystal data	
Chemical formula	C_21_H_20_N_3_^+^·C_24_H_20_B^−^·2CH_3_N
*M*_r_	715.72
Crystal system, space group	Triclinic, *P* 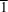
Temperature (K)	90
*a*, *b*, *c* (Å)	13.5215 (13), 13.5537 (14), 13.6079 (14)
α, β, γ (°)	92.202 (4), 113.379 (3), 117.604 (4)
*V* (Å^3^)	1951.3 (3)
*Z*	2
Radiation type	Mo *K*α
μ (mm^−1^)	0.07
Crystal size (mm)	0.30 × 0.30 × 0.15

Data collection	
Diffractometer	Bruker PHOTON II CPAD
Absorption correction	Multi-scan (*SADABS*; Krause *et al.*, 2015 ▸)
*T*_min_, *T*_max_	0.678, 0.746
No. of measured, independent and observed [*I* > 2σ(*I*)] reflections	24904, 9444, 7510
*R*_int_	0.069
(sin θ/λ)_max_ (Å^−1^)	0.724

Refinement	
*R*[*F* ^2^ > 2σ(*F* ^2^)], *wR*(*F* ^2^), *S*	0.060, 0.149, 1.03
No. of reflections	9444
No. of parameters	498
H-atom treatment	H-atom parameters constrained
Δρ_max_, Δρ_min_ (e Å^−3^)	0.32, −0.41

Computer programs: *APEX4* and *SAINT* (Bruker, 2021 ▸), *SIR2019* (Burla *et al.*, 2015 ▸), *SHELXL2018/3* (Sheldrick, 2015 ▸), *DIAMOND* (Brandenburg, 2014 ▸) and *Yadokari-XG* (Kabuto 2009 ▸).

## full crystallographic data

### Crystal data

**Table:**

C_21_H_20_N_3_^+^·C_24_H_20_B^−^·2CH_3_N	*Z* = 2
*M**_r_* = 715.72	*F*(000) = 760
Triclinic, *P*1	*D*_x_ = 1.218 Mg m^−^^3^
*a* = 13.5215 (13) Å	Mo *K*α radiation, λ = 0.71069 Å
*b* = 13.5537 (14) Å	Cell parameters from 5009 reflections
*c* = 13.6079 (14) Å	θ = 2.8–30.7°
α = 92.202 (4)°	µ = 0.07 mm^−^^1^
β = 113.379 (3)°	*T* = 90 K
γ = 117.604 (4)°	Platelet, red
*V* = 1951.3 (3) Å^3^	0.30 × 0.30 × 0.15 mm

### Data collection

**Table:**

Bruker PHOTON II CPAD diffractometer	7510 reflections with *I* > 2σ(*I*)
Radiation source: fine-focus sealed tube	*R*_int_ = 0.069
φ and ω scans	θ_max_ = 31.0°, θ_min_ = 1.7°
Absorption correction: multi-scan (SADABS; Krause et al., 2015)	*h* = −19→19
*T*_min_ = 0.678, *T*_max_ = 0.746	*k* = −17→18
24904 measured reflections	*l* = −18→19
9444 independent reflections	

### Refinement

**Table:**

Refinement on *F*^2^	Primary atom site location: structure-invariant direct methods
Least-squares matrix: full	Hydrogen site location: inferred from neighbouring sites
*R*[*F*^2^ > 2σ(*F*^2^)] = 0.060	H-atom parameters constrained
*wR*(*F*^2^) = 0.149	*w* = 1/[σ^2^(*F*_o_^2^) + (0.0555*P*)^2^ + 0.6357*P*] where *P* = (*F*_o_^2^ + 2*F*_c_^2^)/3
*S* = 1.03	(Δ/σ)_max_ < 0.001
9444 reflections	Δρ_max_ = 0.32 e Å^−^^3^
498 parameters	Δρ_min_ = −0.41 e Å^−^^3^
0 restraints	

### Special details

**Table:**

Geometry. All esds (except the esd in the dihedral angle between two l.s. planes) are estimated using the full covariance matrix. The cell esds are taken into account individually in the estimation of esds in distances, angles and torsion angles; correlations between esds in cell parameters are only used when they are defined by crystal symmetry. An approximate (isotropic) treatment of cell esds is used for estimating esds involving l.s. planes.
Refinement. Refinement of F^2^ against ALL reflections. The weighted R-factor wR and goodness of fit S are based on F^2^, conventional R-factors R are based on F, with F set to zero for negative F^2^. The threshold expression of F^2^ > 2sigma(F^2^) is used only for calculating R-factors(gt) etc. and is not relevant to the choice of reflections for refinement. R-factors based on F^2^ are statistically about twice as large as those based on F, and R- factors based on ALL data will be even larger.All hydrogen atoms are geometrically fixed using a riding-model approximation with C–H = 0.95 (for phenyl), 0.98 (for methyl), 0.99 (for methylene), and N–H = 0.88 Å.

### Fractional atomic coordinates and isotropic or equivalent isotropic displacement parameters (Å^2^)

**Table:**

	*x*	*y*	*z*	*U*_iso_*/*U*_eq_	
C1	0.10283 (13)	0.18417 (12)	0.65451 (11)	0.0151 (3)	
C2	−0.02177 (13)	0.12168 (12)	0.64193 (12)	0.0158 (3)	
C3	−0.05470 (14)	0.03582 (13)	0.69845 (12)	0.0178 (3)	
H3	0.007723	0.021889	0.747351	0.021*	
C4	−0.17723 (14)	−0.02804 (13)	0.68301 (12)	0.0180 (3)	
C5	−0.26821 (14)	−0.00588 (14)	0.60872 (13)	0.0208 (3)	
H5	−0.352926	−0.050353	0.596273	0.025*	
C6	−0.23797 (14)	0.07748 (14)	0.55434 (13)	0.0196 (3)	
H6	−0.301549	0.090190	0.505573	0.024*	
C7	−0.11306 (13)	0.14519 (13)	0.56974 (12)	0.0160 (3)	
C8	−0.07585 (14)	0.23343 (13)	0.51487 (12)	0.0166 (3)	
C9	−0.16161 (15)	0.26139 (13)	0.44044 (13)	0.0203 (3)	
H9	−0.246831	0.221004	0.427140	0.024*	
C10	−0.12532 (16)	0.34412 (14)	0.38777 (13)	0.0221 (3)	
H10	−0.185547	0.359570	0.337438	0.026*	
C11	0.00140 (16)	0.40759 (14)	0.40713 (12)	0.0206 (3)	
C12	0.08861 (15)	0.38404 (13)	0.48036 (12)	0.0202 (3)	
H12	0.174244	0.427117	0.495054	0.024*	
C13	0.05015 (14)	0.29626 (13)	0.53290 (12)	0.0162 (3)	
C14	0.26588 (14)	0.32666 (13)	0.61385 (13)	0.0190 (3)	
H14	0.323686	0.320748	0.682834	0.023*	
H14A	0.296570	0.409724	0.619650	0.023*	
C15	0.26718 (16)	0.27342 (16)	0.51465 (14)	0.0250 (4)	
H15	0.353304	0.314013	0.523789	0.037*	
H15A	0.238071	0.191490	0.509568	0.037*	
H15B	0.210987	0.280359	0.446460	0.037*	
C16	0.19764 (13)	0.15390 (13)	0.72163 (12)	0.0159 (3)	
C17	0.19897 (14)	0.06352 (14)	0.67151 (13)	0.0216 (3)	
H17	0.139450	0.021709	0.595916	0.026*	
C18	0.28669 (15)	0.03388 (15)	0.73113 (14)	0.0237 (3)	
H18	0.287781	−0.027485	0.696375	0.028*	
C19	0.37266 (14)	0.09448 (14)	0.84176 (13)	0.0207 (3)	
H19	0.433250	0.074912	0.882714	0.025*	
C20	0.37059 (14)	0.18335 (13)	0.89275 (13)	0.0198 (3)	
H20	0.429525	0.224226	0.968650	0.024*	
C21	0.28259 (14)	0.21321 (13)	0.83343 (12)	0.0182 (3)	
H21	0.280468	0.273462	0.868848	0.022*	
C22	0.12205 (13)	0.29457 (13)	0.13371 (12)	0.0165 (3)	
C23	0.05756 (14)	0.18979 (13)	0.05175 (12)	0.0186 (3)	
H23	0.087510	0.186105	0.000094	0.022*	
C24	−0.04892 (14)	0.09093 (13)	0.04352 (13)	0.0211 (3)	
H24	−0.090456	0.021982	−0.013482	0.025*	
C25	−0.09401 (14)	0.09344 (14)	0.11858 (13)	0.0218 (3)	
H25	−0.167324	0.026941	0.112436	0.026*	
C26	−0.03077 (14)	0.19422 (14)	0.20276 (13)	0.0203 (3)	
H26	−0.059578	0.196448	0.255658	0.024*	
C27	0.07526 (14)	0.29226 (13)	0.20949 (12)	0.0182 (3)	
H27	0.117457	0.360257	0.267857	0.022*	
C28	0.27575 (14)	0.53038 (13)	0.20596 (12)	0.0165 (3)	
C29	0.39743 (14)	0.62668 (13)	0.27226 (13)	0.0214 (3)	
H29	0.467612	0.618068	0.288191	0.026*	
C30	0.42072 (17)	0.73433 (14)	0.31598 (14)	0.0277 (4)	
H30	0.505085	0.796966	0.360163	0.033*	
C31	0.32062 (18)	0.74988 (15)	0.29491 (15)	0.0285 (4)	
H31	0.335320	0.822368	0.325800	0.034*	
C32	0.19867 (17)	0.65776 (15)	0.22795 (15)	0.0277 (4)	
H32	0.129170	0.667520	0.211816	0.033*	
C33	0.17714 (14)	0.55105 (14)	0.18411 (14)	0.0212 (3)	
H33	0.092598	0.489771	0.137643	0.025*	
C34	0.24620 (13)	0.42353 (12)	0.02563 (12)	0.0156 (3)	
C35	0.13375 (14)	0.37692 (13)	−0.07495 (12)	0.0182 (3)	
H35	0.055675	0.327850	−0.075250	0.022*	
C36	0.13246 (15)	0.39999 (13)	−0.17433 (13)	0.0201 (3)	
H36	0.054400	0.365836	−0.240688	0.024*	
C37	0.24450 (15)	0.47252 (13)	−0.17690 (13)	0.0203 (3)	
H37	0.243851	0.489055	−0.244295	0.024*	
C38	0.35770 (15)	0.52053 (13)	−0.07894 (13)	0.0201 (3)	
H38	0.435400	0.570005	−0.079103	0.024*	
C39	0.35716 (14)	0.49611 (13)	0.01924 (12)	0.0179 (3)	
H39	0.435672	0.530204	0.085129	0.021*	
C40	0.36523 (13)	0.38498 (12)	0.22126 (12)	0.0154 (3)	
C41	0.40842 (14)	0.33088 (13)	0.17341 (12)	0.0182 (3)	
H41	0.374276	0.310812	0.094729	0.022*	
C42	0.49936 (15)	0.30514 (14)	0.23617 (13)	0.0210 (3)	
H42	0.526983	0.269754	0.200185	0.025*	
C43	0.54946 (14)	0.33132 (14)	0.35131 (13)	0.0216 (3)	
H43	0.611657	0.314478	0.394730	0.026*	
C44	0.50703 (14)	0.38249 (13)	0.40172 (13)	0.0200 (3)	
H44	0.539046	0.399432	0.480192	0.024*	
C45	0.41752 (14)	0.40920 (13)	0.33769 (12)	0.0176 (3)	
H45	0.390885	0.445202	0.374305	0.021*	
C46	0.35854 (19)	−0.00009 (19)	0.12689 (16)	0.0409 (5)	
H46	0.288511	−0.079545	0.107370	0.061*	
H46A	0.397972	0.004480	0.079028	0.061*	
H46B	0.326431	0.052423	0.116267	0.061*	
C47	0.45173 (15)	0.03317 (15)	0.24280 (15)	0.0261 (4)	
C48	0.23117 (19)	0.69337 (16)	−0.02061 (17)	0.0344 (4)	
H48	0.284178	0.715801	0.060117	0.052*	
H48A	0.198998	0.611852	−0.051468	0.052*	
H48B	0.160080	0.704240	−0.036650	0.052*	
C49	0.30580 (16)	0.76501 (15)	−0.07085 (14)	0.0267 (4)	
N1	0.13589 (11)	0.26686 (11)	0.60274 (10)	0.0160 (3)	
N2	−0.21233 (13)	−0.10941 (12)	0.73872 (12)	0.0244 (3)	
H2	−0.156112	−0.122643	0.786359	0.029*	
H2A	−0.291077	−0.148392	0.726836	0.029*	
N3	0.03525 (15)	0.49152 (13)	0.35363 (12)	0.0277 (3)	
H3A	0.113866	0.531937	0.365386	0.033*	
H3B	−0.021640	0.504984	0.307539	0.033*	
B1	0.25161 (15)	0.40776 (14)	0.14659 (13)	0.0148 (3)	
N4	0.52247 (15)	0.05982 (14)	0.33369 (14)	0.0347 (4)	
N5	0.36569 (16)	0.82227 (15)	−0.10868 (14)	0.0370 (4)	

### Atomic displacement parameters (Å^2^)

**Table:**

	*U*^11^	*U*^22^	*U*^33^	*U*^12^	*U*^13^	*U*^23^
C1	0.0162 (7)	0.0135 (7)	0.0130 (6)	0.0067 (6)	0.0060 (6)	0.0022 (5)
C2	0.0156 (6)	0.0140 (7)	0.0153 (7)	0.0070 (6)	0.0060 (6)	0.0023 (5)
C3	0.0158 (7)	0.0159 (7)	0.0185 (7)	0.0073 (6)	0.0065 (6)	0.0051 (6)
C4	0.0179 (7)	0.0149 (7)	0.0182 (7)	0.0061 (6)	0.0088 (6)	0.0036 (6)
C5	0.0167 (7)	0.0217 (8)	0.0227 (8)	0.0086 (6)	0.0098 (6)	0.0049 (6)
C6	0.0168 (7)	0.0230 (8)	0.0189 (7)	0.0111 (6)	0.0074 (6)	0.0053 (6)
C7	0.0174 (7)	0.0152 (7)	0.0149 (7)	0.0087 (6)	0.0071 (6)	0.0022 (5)
C8	0.0186 (7)	0.0152 (7)	0.0151 (7)	0.0095 (6)	0.0066 (6)	0.0024 (6)
C9	0.0198 (7)	0.0190 (7)	0.0193 (7)	0.0112 (6)	0.0055 (6)	0.0029 (6)
C10	0.0279 (8)	0.0210 (8)	0.0178 (7)	0.0160 (7)	0.0072 (7)	0.0058 (6)
C11	0.0302 (8)	0.0180 (7)	0.0161 (7)	0.0139 (7)	0.0115 (7)	0.0055 (6)
C12	0.0225 (7)	0.0188 (7)	0.0196 (7)	0.0095 (6)	0.0115 (6)	0.0064 (6)
C13	0.0196 (7)	0.0151 (7)	0.0130 (6)	0.0096 (6)	0.0063 (6)	0.0027 (5)
C14	0.0151 (7)	0.0201 (7)	0.0197 (7)	0.0068 (6)	0.0090 (6)	0.0077 (6)
C15	0.0249 (8)	0.0330 (9)	0.0246 (8)	0.0170 (7)	0.0157 (7)	0.0107 (7)
C16	0.0139 (6)	0.0157 (7)	0.0181 (7)	0.0067 (5)	0.0085 (6)	0.0063 (6)
C17	0.0185 (7)	0.0212 (8)	0.0189 (7)	0.0092 (6)	0.0052 (6)	0.0004 (6)
C18	0.0234 (8)	0.0239 (8)	0.0259 (8)	0.0154 (7)	0.0101 (7)	0.0035 (7)
C19	0.0170 (7)	0.0224 (8)	0.0239 (8)	0.0118 (6)	0.0086 (6)	0.0087 (6)
C20	0.0164 (7)	0.0192 (7)	0.0188 (7)	0.0076 (6)	0.0057 (6)	0.0058 (6)
C21	0.0181 (7)	0.0153 (7)	0.0183 (7)	0.0079 (6)	0.0072 (6)	0.0036 (6)
C22	0.0141 (6)	0.0164 (7)	0.0180 (7)	0.0081 (6)	0.0064 (6)	0.0065 (6)
C23	0.0186 (7)	0.0184 (7)	0.0178 (7)	0.0087 (6)	0.0086 (6)	0.0053 (6)
C24	0.0177 (7)	0.0156 (7)	0.0221 (7)	0.0060 (6)	0.0060 (6)	0.0035 (6)
C25	0.0150 (7)	0.0192 (8)	0.0266 (8)	0.0062 (6)	0.0088 (6)	0.0099 (6)
C26	0.0189 (7)	0.0225 (8)	0.0234 (8)	0.0114 (6)	0.0123 (7)	0.0104 (6)
C27	0.0183 (7)	0.0174 (7)	0.0178 (7)	0.0086 (6)	0.0084 (6)	0.0053 (6)
C28	0.0188 (7)	0.0168 (7)	0.0166 (7)	0.0088 (6)	0.0110 (6)	0.0077 (6)
C29	0.0190 (7)	0.0188 (8)	0.0231 (8)	0.0093 (6)	0.0078 (6)	0.0037 (6)
C30	0.0289 (8)	0.0180 (8)	0.0281 (8)	0.0088 (7)	0.0110 (7)	0.0028 (7)
C31	0.0402 (10)	0.0198 (8)	0.0346 (9)	0.0172 (8)	0.0234 (8)	0.0097 (7)
C32	0.0340 (9)	0.0286 (9)	0.0421 (10)	0.0224 (8)	0.0284 (9)	0.0199 (8)
C33	0.0186 (7)	0.0196 (8)	0.0291 (8)	0.0093 (6)	0.0146 (7)	0.0113 (6)
C34	0.0172 (7)	0.0133 (7)	0.0181 (7)	0.0085 (6)	0.0090 (6)	0.0054 (5)
C35	0.0180 (7)	0.0162 (7)	0.0208 (7)	0.0088 (6)	0.0094 (6)	0.0069 (6)
C36	0.0226 (7)	0.0188 (7)	0.0171 (7)	0.0122 (6)	0.0063 (6)	0.0048 (6)
C37	0.0316 (8)	0.0189 (7)	0.0189 (7)	0.0164 (7)	0.0151 (7)	0.0093 (6)
C38	0.0219 (7)	0.0175 (7)	0.0243 (8)	0.0091 (6)	0.0149 (7)	0.0085 (6)
C39	0.0169 (7)	0.0165 (7)	0.0180 (7)	0.0074 (6)	0.0079 (6)	0.0044 (6)
C40	0.0147 (6)	0.0116 (6)	0.0192 (7)	0.0050 (5)	0.0092 (6)	0.0056 (5)
C41	0.0211 (7)	0.0187 (7)	0.0171 (7)	0.0100 (6)	0.0112 (6)	0.0068 (6)
C42	0.0245 (8)	0.0207 (8)	0.0265 (8)	0.0142 (6)	0.0164 (7)	0.0102 (6)
C43	0.0184 (7)	0.0213 (8)	0.0256 (8)	0.0112 (6)	0.0092 (7)	0.0103 (6)
C44	0.0183 (7)	0.0193 (7)	0.0176 (7)	0.0080 (6)	0.0061 (6)	0.0063 (6)
C45	0.0187 (7)	0.0156 (7)	0.0186 (7)	0.0080 (6)	0.0100 (6)	0.0038 (6)
C46	0.0347 (10)	0.0394 (11)	0.0310 (10)	0.0191 (9)	0.0014 (9)	−0.0018 (9)
C47	0.0193 (7)	0.0234 (8)	0.0353 (10)	0.0121 (7)	0.0113 (8)	0.0083 (7)
C48	0.0413 (10)	0.0282 (9)	0.0399 (10)	0.0185 (8)	0.0236 (9)	0.0132 (8)
C49	0.0274 (8)	0.0283 (9)	0.0222 (8)	0.0168 (7)	0.0072 (7)	0.0040 (7)
N1	0.0146 (6)	0.0159 (6)	0.0151 (6)	0.0068 (5)	0.0063 (5)	0.0036 (5)
N2	0.0199 (6)	0.0234 (7)	0.0297 (7)	0.0095 (6)	0.0131 (6)	0.0137 (6)
N3	0.0381 (8)	0.0284 (8)	0.0284 (7)	0.0221 (7)	0.0193 (7)	0.0173 (6)
B1	0.0133 (7)	0.0148 (7)	0.0153 (7)	0.0062 (6)	0.0072 (6)	0.0042 (6)
N4	0.0264 (8)	0.0353 (9)	0.0360 (9)	0.0156 (7)	0.0093 (7)	0.0132 (7)
N5	0.0351 (9)	0.0424 (10)	0.0347 (8)	0.0198 (8)	0.0176 (8)	0.0133 (8)

### Geometric parameters (Å, º)

**Table:**

C1—N1	1.3411 (19)	C26—H26	0.9500
C1—C2	1.422 (2)	C27—H27	0.9500
C1—C16	1.494 (2)	C28—C29	1.402 (2)
C2—C3	1.416 (2)	C28—C33	1.408 (2)
C2—C7	1.420 (2)	C28—B1	1.645 (2)
C3—C4	1.385 (2)	C29—C30	1.393 (2)
C3—H3	0.9500	C29—H29	0.9500
C4—N2	1.372 (2)	C30—C31	1.385 (3)
C4—C5	1.416 (2)	C30—H30	0.9500
C5—C6	1.367 (2)	C31—C32	1.386 (3)
C5—H5	0.9500	C31—H31	0.9500
C6—C7	1.417 (2)	C32—C33	1.392 (2)
C6—H6	0.9500	C32—H32	0.9500
C7—C8	1.432 (2)	C33—H33	0.9500
C8—C13	1.413 (2)	C34—C39	1.403 (2)
C8—C9	1.421 (2)	C34—C35	1.406 (2)
C9—C10	1.357 (2)	C34—B1	1.645 (2)
C9—H9	0.9500	C35—C36	1.395 (2)
C10—C11	1.416 (2)	C35—H35	0.9500
C10—H10	0.9500	C36—C37	1.388 (2)
C11—N3	1.371 (2)	C36—H36	0.9500
C11—C12	1.388 (2)	C37—C38	1.391 (2)
C12—C13	1.409 (2)	C37—H37	0.9500
C12—H12	0.9500	C38—C39	1.391 (2)
C13—N1	1.4103 (19)	C38—H38	0.9500
C14—N1	1.4929 (19)	C39—H39	0.9500
C14—C15	1.516 (2)	C40—C41	1.402 (2)
C14—H14	0.9900	C40—C45	1.405 (2)
C14—H14A	0.9900	C40—B1	1.647 (2)
C15—H15	0.9800	C41—C42	1.397 (2)
C15—H15A	0.9800	C41—H41	0.9500
C15—H15B	0.9800	C42—C43	1.390 (2)
C16—C17	1.390 (2)	C42—H42	0.9500
C16—C21	1.394 (2)	C43—C44	1.388 (2)
C17—C18	1.389 (2)	C43—H43	0.9500
C17—H17	0.9500	C44—C45	1.396 (2)
C18—C19	1.387 (2)	C44—H44	0.9500
C18—H18	0.9500	C45—H45	0.9500
C19—C20	1.384 (2)	C46—C47	1.456 (3)
C19—H19	0.9500	C46—H46	0.9800
C20—C21	1.393 (2)	C46—H46A	0.9800
C20—H20	0.9500	C46—H46B	0.9800
C21—H21	0.9500	C47—N4	1.132 (2)
C22—C27	1.402 (2)	C48—C49	1.456 (3)
C22—C23	1.407 (2)	C48—H48	0.9800
C22—B1	1.643 (2)	C48—H48A	0.9800
C23—C24	1.398 (2)	C48—H48B	0.9800
C23—H23	0.9500	C49—N5	1.141 (2)
C24—C25	1.388 (2)	N2—H2	0.8800
C24—H24	0.9500	N2—H2A	0.8800
C25—C26	1.389 (2)	N3—H3A	0.8800
C25—H25	0.9500	N3—H3B	0.8800
C26—C27	1.397 (2)		
			
N1—C1—C2	121.19 (13)	C26—C27—H27	118.6
N1—C1—C16	118.78 (12)	C22—C27—H27	118.6
C2—C1—C16	119.96 (13)	C29—C28—C33	114.76 (14)
C3—C2—C7	120.71 (13)	C29—C28—B1	122.18 (13)
C3—C2—C1	120.51 (13)	C33—C28—B1	122.68 (13)
C7—C2—C1	118.76 (14)	C30—C29—C28	123.39 (15)
C4—C3—C2	120.53 (14)	C30—C29—H29	118.3
C4—C3—H3	119.7	C28—C29—H29	118.3
C2—C3—H3	119.7	C31—C30—C29	119.88 (16)
N2—C4—C3	122.46 (14)	C31—C30—H30	120.1
N2—C4—C5	119.23 (13)	C29—C30—H30	120.1
C3—C4—C5	118.29 (14)	C30—C31—C32	118.79 (16)
C6—C5—C4	122.05 (14)	C30—C31—H31	120.6
C6—C5—H5	119.0	C32—C31—H31	120.6
C4—C5—H5	119.0	C31—C32—C33	120.57 (15)
C5—C6—C7	120.87 (14)	C31—C32—H32	119.7
C5—C6—H6	119.6	C33—C32—H32	119.7
C7—C6—H6	119.6	C32—C33—C28	122.58 (15)
C6—C7—C2	117.53 (14)	C32—C33—H33	118.7
C6—C7—C8	123.39 (14)	C28—C33—H33	118.7
C2—C7—C8	119.07 (13)	C39—C34—C35	115.19 (14)
C13—C8—C9	117.15 (14)	C39—C34—B1	120.42 (12)
C13—C8—C7	120.24 (13)	C35—C34—B1	124.11 (13)
C9—C8—C7	122.61 (14)	C36—C35—C34	122.49 (14)
C10—C9—C8	121.91 (15)	C36—C35—H35	118.8
C10—C9—H9	119.0	C34—C35—H35	118.8
C8—C9—H9	119.0	C37—C36—C35	120.44 (14)
C9—C10—C11	120.63 (15)	C37—C36—H36	119.8
C9—C10—H10	119.7	C35—C36—H36	119.8
C11—C10—H10	119.7	C36—C37—C38	118.73 (14)
N3—C11—C12	121.56 (15)	C36—C37—H37	120.6
N3—C11—C10	119.15 (15)	C38—C37—H37	120.6
C12—C11—C10	119.29 (15)	C39—C38—C37	120.01 (14)
C11—C12—C13	120.01 (14)	C39—C38—H38	120.0
C11—C12—H12	120.0	C37—C38—H38	120.0
C13—C12—H12	120.0	C38—C39—C34	123.13 (14)
C12—C13—N1	120.74 (13)	C38—C39—H39	118.4
C12—C13—C8	120.97 (14)	C34—C39—H39	118.4
N1—C13—C8	118.27 (13)	C41—C40—C45	115.24 (13)
N1—C14—C15	110.96 (12)	C41—C40—B1	122.78 (13)
N1—C14—H14	109.4	C45—C40—B1	121.73 (13)
C15—C14—H14	109.4	C42—C41—C40	122.97 (14)
N1—C14—H14A	109.4	C42—C41—H41	118.5
C15—C14—H14A	109.4	C40—C41—H41	118.5
H14—C14—H14A	108.0	C43—C42—C41	119.98 (14)
C14—C15—H15	109.5	C43—C42—H42	120.0
C14—C15—H15A	109.5	C41—C42—H42	120.0
H15—C15—H15A	109.5	C44—C43—C42	118.84 (14)
C14—C15—H15B	109.5	C44—C43—H43	120.6
H15—C15—H15B	109.5	C42—C43—H43	120.6
H15A—C15—H15B	109.5	C43—C44—C45	120.31 (14)
C17—C16—C21	119.83 (14)	C43—C44—H44	119.8
C17—C16—C1	118.98 (13)	C45—C44—H44	119.8
C21—C16—C1	121.18 (13)	C44—C45—C40	122.63 (14)
C18—C17—C16	120.48 (14)	C44—C45—H45	118.7
C18—C17—H17	119.8	C40—C45—H45	118.7
C16—C17—H17	119.8	C47—C46—H46	109.5
C19—C18—C17	119.55 (15)	C47—C46—H46A	109.5
C19—C18—H18	120.2	H46—C46—H46A	109.5
C17—C18—H18	120.2	C47—C46—H46B	109.5
C20—C19—C18	120.29 (14)	H46—C46—H46B	109.5
C20—C19—H19	119.9	H46A—C46—H46B	109.5
C18—C19—H19	119.9	N4—C47—C46	178.3 (2)
C19—C20—C21	120.39 (14)	C49—C48—H48	109.5
C19—C20—H20	119.8	C49—C48—H48A	109.5
C21—C20—H20	119.8	H48—C48—H48A	109.5
C20—C21—C16	119.43 (14)	C49—C48—H48B	109.5
C20—C21—H21	120.3	H48—C48—H48B	109.5
C16—C21—H21	120.3	H48A—C48—H48B	109.5
C27—C22—C23	115.56 (14)	N5—C49—C48	179.0 (2)
C27—C22—B1	122.38 (13)	C1—N1—C13	122.40 (12)
C23—C22—B1	121.80 (13)	C1—N1—C14	119.54 (12)
C24—C23—C22	122.44 (14)	C13—N1—C14	117.99 (12)
C24—C23—H23	118.8	C4—N2—H2	120.0
C22—C23—H23	118.8	C4—N2—H2A	120.0
C25—C24—C23	120.04 (14)	H2—N2—H2A	120.0
C25—C24—H24	120.0	C11—N3—H3A	120.0
C23—C24—H24	120.0	C11—N3—H3B	120.0
C24—C25—C26	119.28 (14)	H3A—N3—H3B	120.0
C24—C25—H25	120.4	C22—B1—C28	112.62 (12)
C26—C25—H25	120.4	C22—B1—C34	112.51 (12)
C25—C26—C27	119.86 (15)	C28—B1—C34	103.66 (12)
C25—C26—H26	120.1	C22—B1—C40	104.63 (12)
C27—C26—H26	120.1	C28—B1—C40	111.81 (12)
C26—C27—C22	122.77 (14)	C34—B1—C40	111.84 (12)
			
N1—C1—C2—C3	−179.05 (13)	C28—C29—C30—C31	0.3 (3)
C16—C1—C2—C3	4.1 (2)	C29—C30—C31—C32	−1.6 (3)
N1—C1—C2—C7	2.4 (2)	C30—C31—C32—C33	1.0 (3)
C16—C1—C2—C7	−174.45 (13)	C31—C32—C33—C28	0.9 (3)
C7—C2—C3—C4	1.0 (2)	C29—C28—C33—C32	−2.0 (2)
C1—C2—C3—C4	−177.60 (13)	B1—C28—C33—C32	−175.08 (15)
C2—C3—C4—N2	−177.87 (14)	C39—C34—C35—C36	0.7 (2)
C2—C3—C4—C5	0.6 (2)	B1—C34—C35—C36	174.57 (14)
N2—C4—C5—C6	177.17 (14)	C34—C35—C36—C37	−0.9 (2)
C3—C4—C5—C6	−1.4 (2)	C35—C36—C37—C38	0.7 (2)
C4—C5—C6—C7	0.5 (2)	C36—C37—C38—C39	−0.4 (2)
C5—C6—C7—C2	1.1 (2)	C37—C38—C39—C34	0.3 (2)
C5—C6—C7—C8	179.81 (14)	C35—C34—C39—C38	−0.4 (2)
C3—C2—C7—C6	−1.8 (2)	B1—C34—C39—C38	−174.51 (14)
C1—C2—C7—C6	176.79 (13)	C45—C40—C41—C42	−1.6 (2)
C3—C2—C7—C8	179.41 (13)	B1—C40—C41—C42	−176.04 (14)
C1—C2—C7—C8	−2.0 (2)	C40—C41—C42—C43	1.2 (2)
C6—C7—C8—C13	−178.95 (13)	C41—C42—C43—C44	0.3 (2)
C2—C7—C8—C13	−0.2 (2)	C42—C43—C44—C45	−1.3 (2)
C6—C7—C8—C9	0.5 (2)	C43—C44—C45—C40	0.9 (2)
C2—C7—C8—C9	179.27 (13)	C41—C40—C45—C44	0.6 (2)
C13—C8—C9—C10	0.5 (2)	B1—C40—C45—C44	175.05 (13)
C7—C8—C9—C10	−179.00 (14)	C2—C1—N1—C13	−0.4 (2)
C8—C9—C10—C11	−1.2 (2)	C16—C1—N1—C13	176.45 (12)
C9—C10—C11—N3	−179.20 (14)	C2—C1—N1—C14	−177.17 (12)
C9—C10—C11—C12	0.3 (2)	C16—C1—N1—C14	−0.33 (19)
N3—C11—C12—C13	−179.28 (14)	C12—C13—N1—C1	179.70 (13)
C10—C11—C12—C13	1.2 (2)	C8—C13—N1—C1	−1.9 (2)
C11—C12—C13—N1	176.47 (13)	C12—C13—N1—C14	−3.5 (2)
C11—C12—C13—C8	−1.9 (2)	C8—C13—N1—C14	174.94 (12)
C9—C8—C13—C12	1.0 (2)	C15—C14—N1—C1	97.48 (16)
C7—C8—C13—C12	−179.44 (13)	C15—C14—N1—C13	−79.45 (16)
C9—C8—C13—N1	−177.37 (13)	C27—C22—B1—C28	−30.72 (19)
C7—C8—C13—N1	2.2 (2)	C23—C22—B1—C28	155.40 (13)
N1—C1—C16—C17	−93.33 (17)	C27—C22—B1—C34	−147.44 (14)
C2—C1—C16—C17	83.56 (18)	C23—C22—B1—C34	38.68 (19)
N1—C1—C16—C21	87.83 (18)	C27—C22—B1—C40	90.93 (16)
C2—C1—C16—C21	−95.29 (17)	C23—C22—B1—C40	−82.95 (16)
C21—C16—C17—C18	−1.8 (2)	C29—C28—B1—C22	148.75 (14)
C1—C16—C17—C18	179.33 (14)	C33—C28—B1—C22	−38.68 (19)
C16—C17—C18—C19	0.6 (3)	C29—C28—B1—C34	−89.37 (16)
C17—C18—C19—C20	0.5 (2)	C33—C28—B1—C34	83.20 (16)
C18—C19—C20—C21	−0.3 (2)	C29—C28—B1—C40	31.28 (19)
C19—C20—C21—C16	−0.9 (2)	C33—C28—B1—C40	−156.15 (14)
C17—C16—C21—C20	2.0 (2)	C39—C34—B1—C22	−164.01 (13)
C1—C16—C21—C20	−179.19 (14)	C35—C34—B1—C22	22.4 (2)
C27—C22—C23—C24	2.3 (2)	C39—C34—B1—C28	74.04 (16)
B1—C22—C23—C24	176.53 (14)	C35—C34—B1—C28	−99.51 (16)
C22—C23—C24—C25	−0.7 (2)	C39—C34—B1—C40	−46.58 (18)
C23—C24—C25—C26	−1.3 (2)	C35—C34—B1—C40	139.86 (14)
C24—C25—C26—C27	1.4 (2)	C41—C40—B1—C22	93.19 (16)
C25—C26—C27—C22	0.3 (2)	C45—C40—B1—C22	−80.86 (16)
C23—C22—C27—C26	−2.1 (2)	C41—C40—B1—C28	−144.63 (14)
B1—C22—C27—C26	−176.30 (14)	C45—C40—B1—C28	41.32 (18)
C33—C28—C29—C30	1.4 (2)	C41—C40—B1—C34	−28.87 (19)
B1—C28—C29—C30	174.54 (15)	C45—C40—B1—C34	157.08 (13)

### Hydrogen-bond geometry (Å, º)

*Cg*1–*Cg*5 and *Cg*8 are the centroids of the C22–C27, C28–C33, C34–C39, C40–C45, N1/C1/C2/C7/C8/C13 and C16–C21 rings, respectively.

**Table:**

*D*—H···*A*	*D*—H	H···*A*	*D*···*A*	*D*—H···*A*
N2—H2···*Cg*1^i^	0.88	2.45	3.303 (2)	165
N2—H2*A*···*Cg*4^i^	0.88	2.62	3.3297 (18)	139
C21—H21···*Cg*3^ii^	0.95	2.75	3.685 (2)	167
C25—H25···*Cg*8^i^	0.95	2.79	3.632 (2)	148
C36—H36···*Cg*5^iii^	0.95	2.81	3.3593 (18)	118
C48—H48···*Cg*2	0.98	2.78	3.609 (2)	143
C48—H48*A*···*Cg*3	0.99	2.61	3.488 (2)	149

Symmetry codes: (i) −*x*, −*y*, −*z*+1; (ii) *x*, *y*, *z*+1; (iii) *x*, *y*, *z*−1.
